# An improved phage-display panning method to produce an HM-1 killer toxin anti-idiotypic antibody

**DOI:** 10.1186/1472-6750-9-99

**Published:** 2009-12-14

**Authors:** M Enamul Kabir, Senthilkumar Krishnaswamy, Masahiko Miyamoto, Yasuhiro Furuichi, Tadazumi Komiyama

**Affiliations:** 1 Department of Biochemistry, Faculty of Pharmaceutical Sciences, Niigata University of Pharmacy and Applied Life Sciences, 265-1 Higashijima, Niigata 956-8603, Japan; 2 GeneCare Research Institute Co. Ltd., 200 Kajiwara, Kamakura 247-0063, Japan

## Abstract

**Background:**

Phage-display panning is an integral part of biomedical research. Regular panning methods are sometimes complicated by inefficient detachment of the captured phages from the antigen-coated solid supports, which prompted us to modify. Here, we produce an efficient antigen-specific single chain fragment variable (scFv) antibody by using a target-related molecule that favored selection ofrecombinant antibodies.

**Results:**

To produce more selective and specific anti-idiotypic scFv-antibodies from a cDNA library, constructed from HM-1 killer toxin (HM-1)-neutralizing monoclonal antibodies (nmAb-KT), the method was modified by using an elution buffer supplemented with HM-1 that shares structural and functional similarities with the active site of the scFv antibody. Competitive binding of HM-1 to nmAb-KT allowed easy and quick dissociation of scFv-displayed phages from immobilized nmAb-KT to select specific anti-idiotypic scFv antibodies of HM-1. After modified panning, 80% clones (40/50) showed several times higher binding affinity to nmAb-KT than regular panning. The major populations (48%) of these clones (scFv K1) were genotypically same and had strong cytocidal activity against *Saccharomyces *and *Candida *species. The scFv K1 (*K*_d _value = 4.62 × 10^-8 ^M) had strong reactivity toward nmAb-KT, like HM-1 (*K*_d _value = 6.74 × 10^-9 ^M) as judged by SPR analysis.

**Conclusion:**

The scFv antibodies generated after modified subtractive panning appear to have superior binding properties and cytocidal activity than regular panning. A simple modification of the elution condition in the phage-display panning protocol makes a large difference in determining success. Our method offers an attractive platform to discover potential therapeutic candidates.

## Background

Fungal infections have recently emerged as a growing threat to human health, especially in persons whose immune systems are compromised [1]. Nowadays, the most common treatment for fungal infections is based on the use of amphotericin B, 5-flucytosine and fluconazole. However, these drugs have repeatedly failed against infections caused by *Candida *and *Cryptococcus *species [2]. A series of antigenic peptides from fungal pathogens have been identified that can generate immune responses, and which may assist in developing an antifungal vaccine [3,4]. Therefore, development of novel molecules and alternative therapeutic strategies for the battle against fungal infections is becoming a topical and widely recognized need. To combat against fungal diseases, we are trying to produce a single, but general, antifungal vaccine by using an improved and optimized phage-display panning method that elicits immune responses in immunocompromised individuals who are at risk of invasive fungal infections by opportunistic fungal pathogens, as well as other multiple fungal infections.

One of the most widely used library methods is based on the use of a filamentous phage, a virus that lives on *Escherichia coli*. Using a phage-display system, various antibody fragments can be displayed on the surface of filamentous phages containing the antibody gene as a phagemid [5-8]. However, antibody fragments, including single chain fragment variable (scFv) molecules, have been developed for potential clinical applications [9]. Production of recombinant antibodies using phage-display technology has many advantages over conventional polyclonal and monoclonal antibody production [10-12]. Phage-display technology and antibody engineering have been used to isolate scFvs that can interact with a wide variety of antigens [13,14]. An effective and reliable method is needed to produce antibodies with high antigenic specificity and affinity. In this study, we investigated the de novo selection of DNA that encodes scFvs specific to the immunogen, HM-1 killer toxin (HM-1)-neutralizing monoclonal antibodies (nmAb-KT) from splenocytes of a hyper-immunized mouse.

Conventional methods of phage-display panning immobilize purified antigen to plates or other solid supports to which libraries are applied, and then use extensive washings in detergent-supplemented buffers to select specific phage antibodies [15]. These methods sometimes are not so effective due to tight antigen-antibody binding and attachment to the solid support of the tubes or plates. To optimize the method of phage-display panning and also to recover high-affinity anti-idiotypic phage antibodies from immobilized antigens, different elution conditions have been used [16,17]. Each antigen-antibody interaction requires careful fine-tuning of elution conditions to produce antigen-specific scFv antibodies [17,18]. We are trying to establish an improved, optimized subtractive panning method to select antigen-specific antibodies more efficiently.

Killer toxins are protein molecules that disrupt cell functions in a number of ways, some by making ion channels in cell membranes and others by interacting with membrane channels or receptors or both [19,20]. Among yeast killer toxins, HM-1 is highly stable against heat treatment and a wide range of pH values (pH 2 to 11) and also exhibits the wide spectrum of anti-microbial activity [21-24]. These unique properties of this toxin have made it attractive for therapeutic applications, but not for clinical use because of their instability in the host physiological conditions as well as their antigenicity. To overcome the intrinsic toxicity and chemical instability of HM-1, moreover taking advantage of its immunogenicity, nmAb-KT has been produced. To produce a new anti-fungal drug, previously we used nmAb-KT as an immunogen in mice to get an antigen-specific recombinant anti-idiotypic scFv antibody, sharing structural or functional or both similarities with the active site of HM-1 [3]. In this study, selection of phage-displayed scFv was based on specific antigen nmAb-KT coated on to immunotubes and that was chosen to explore the potential of the cDNA library, constructed from nmAb-KT, to isolate antigen-specific antibody fragments. The strategy applied for the modified subtractive panning, tested within five cycles, was based on the premise that competitive binding of HM-1 to coated nmAb-KT can improve selection efficiency by an easy and quick dissociation of scFv-displayed phages from the immobilized surface. The ultimate goal of this study was to produce recombinant antibodies and to characterize their properties by immunoassay parameters, DNA analysis, product stability and functionality.

## Results

### Selection of clones with improved characteristics

**Figure 1:**
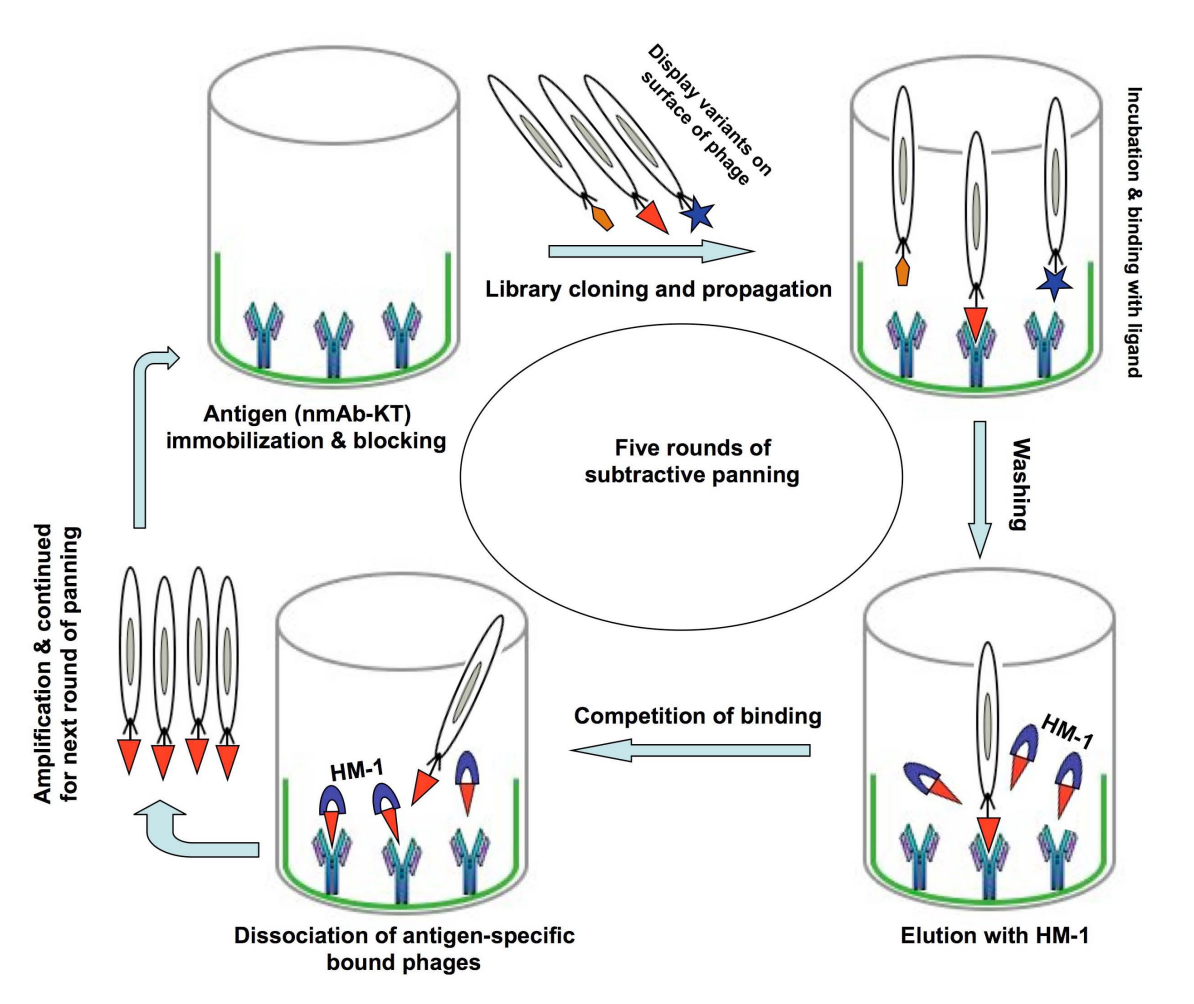
**Schematic representation of the phage-display selection procedure: modified subtractive panning**. Phage particles displaying the members of the library were produced. The specific binder (nmAb-KT) was allowed to bind with the target, and other variants were removed by washing. Molecular variants with specificity for the target were retrieved after multiple cycles of selection and were characterized in detail. Bound phages were eluted with HM-1 containing phosphate buffer (pH 7.0). HM-1 had high binding affinity to the immobilized nmAb-KT. This competition of binding favored quick dissociation of scFv-containing phages from the bound nmAb-KT and consequently increased the elution stringency of the infected phages.

**Figure 2:**
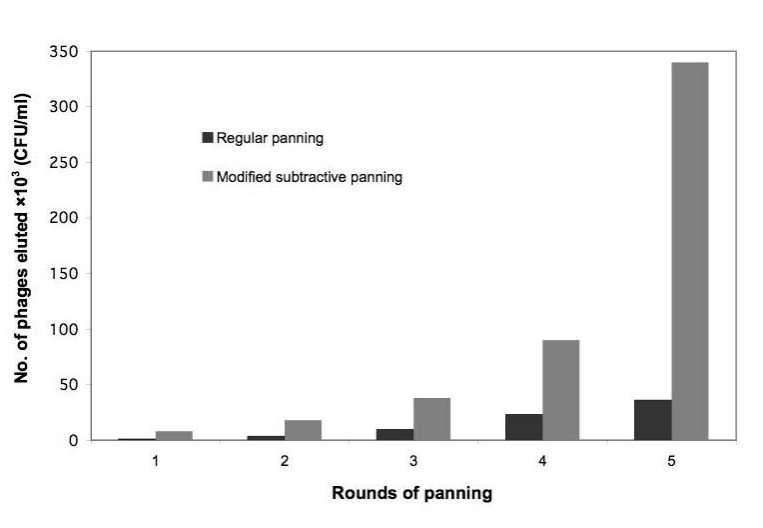
**Enrichment of phage-displayed scFvs by panning**. Phage-displayed scFvs were selected against nmAb-KT and were reinfected into *E. coli *TG1. The number of phages eluted after each round of panning was counted based on the number of colonies (CFU/ml) formed after reinfection of the eluted phage particles in the host bacteria. The results were means of triplicates.

Solid phase panning as described by Griffiths et al. [7] was used to select nmAb-KT-specific phage scFv. Selection of phages with specificity for nmAb-KT was based on increasing stringency of selection at each round of panning (Figure 1). Enrichment was determined to check the number of phages recaptured after each round of panning by counting the colony forming units (CFU) of the infected *E. coli *TG1. The phage-display library consisted of 2.8 × 10^10 ^clones (CFU/ml) with a final diversity of 3.4 × 10^5 ^separate clones after fifth round of modified subtractive panning. Figure 2 showed the phage recovery after each round of regular panning and modified subtractive panning. Transformed *E. coli *TG1 cells in which the amber stop codon between the E-tag DNA sequence and g3 gene is read through, allowing the production of the scFv-E tag-g3 fusion protein. In the presence of helper phage M13KO7, scFv fusion products were displayed on the recombinant phage tips, allowing affinity selection. In the regular panning method, small differences were in the stringency of selection between different rounds of panning, but in modified subtractive panning after two or three rounds of selection, the antigen-binding clones showed a clear enrichment (Figure 2). In modified subtractive panning, the stringency of panning was significantly increased after the fourth and fifth rounds of panning. During different selection rounds of modified subtractive panning, bound scFv-containing phages were eluted easily and quickly from the immobilized nmAb-KT by using an elution buffer supplemented with HM-1 at concentration 10 μg/ml. The phage enrichment for regular panning was not as impressive as for modified subtractive panning. For regular panning, the number of recaptured phages increased approximately 2-fold with low binding affinity in every round, but in modified subtractive panning, the number of recaptured phages increased 3 - 5 fold in the first three rounds of panning but increased 10-fold in the fourth and fifth rounds with higher binding strength to the antigen (Figure 2). Thus, the overall enrichment for modified subtractive panning was approximately 10-fold higher than for regular panning after the final fifth round of panning (Figure 2).

**Figure 3:**
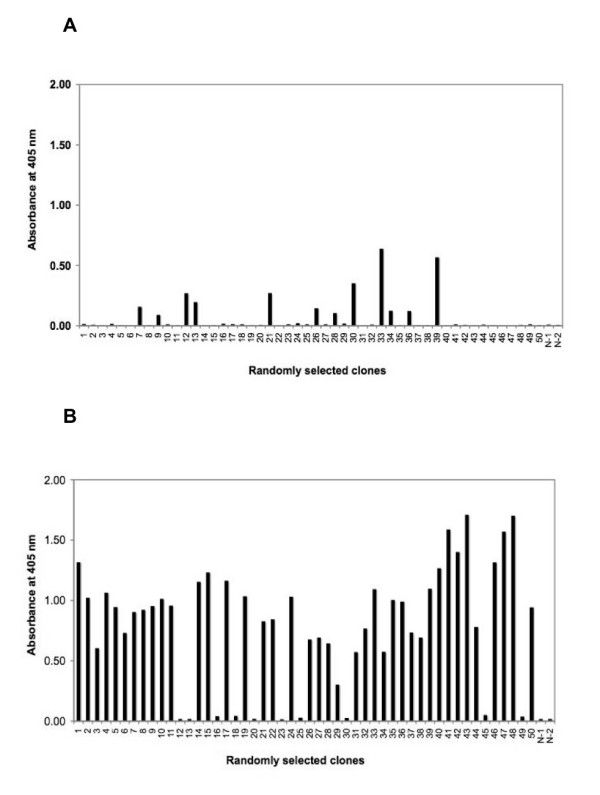
**Identification of positive clones to immobilized antigen**. The binding properties of randomly selected clones to nmAb-KT were measured by microtiter plate ELISA. The supernatant containing soluble scFv antibodies was added to each well of a microtiter plate that had been pre-coated with nmAb-KT and detection was done by HRP-conjugated anti-E-tag antibody. **A. Regular panning**: 12 of 50 (24%) tested clones of infected *E. coli *TG1 contained the anti-idiotypic scFv gene of HM-1 killer toxin, and so soluble scFvs showed positive ELISA signals after the fifth round of panning. **B. Modified subtractive panning**: 40 of 50 (80%) tested clones showed positive ELISA signals after the fifth round of modified subtractive panning. Negative controls consisted of microtiter plates coated with blocking buffer (N-1) or washing buffer (N-2). The results were means of duplicates.

To verify the panning performance, we identified antigen-specific positive clones binding to the immobilized nmAb-KT antigen. The binding properties of 50 randomly selected clones binding to nmAb-KT for both types of panning were measured by using microtiter plate enzyme-linked immunosorbent assay (ELISA). The supernatant containing soluble scFv antibodies was added to each well of a microtiter plate that had been pre-coated with nmAb-KT, and detection was done by using horseradish peroxidase (HRP)-conjugated anti-E-tag antiboy. For regular panning, 24% (12 of 50) tested clones of the infected *E. coli *TG1 contained the anti-idiotypic scFv gene of HM-1 killer toxin, and so soluble scFv showed positive ELISA signals after the fifth round of panning (Figure 3A and Table 1). Interestingly, for modified subtractive panning, 80% (40 of 50) tested clones showed positive ELISA signal, also after the fifth round of panning with higher binding strength to nmAb-KT (Figure 3B and Table 2). Positive binding affinity was defined as an absorbance for immobilized nmAb-KT that was 2-fold above the background or negative controls. Negative controls consisted of microtiter plates coated with blocking buffer (N-1) or washing buffer (N-2).

**Table 1:** Panning results of the phage-displayed library

Antigen	Panning type	Rounds	Positives*
nmAb-KT	Regular panning	5	12/50, 24%
nmAb-KT	Modified subtractive panning	5	40/50, 80%

* Randomly selected clones were grown overnight and supernatants were tested for binding to the immobilized antigen by ELISA.

**Table 2:** Percentages of positive clones based on distinct DNA sequences after the fifth round of modified subtractive panning

Clone type	No. of clones with identical sequence	% of distinct DNA sequences
K1	19/40	48
K2	15/40	38
K3	4/40	10
K4	1/40	2
K5	1/40	2

**Figure 4:**
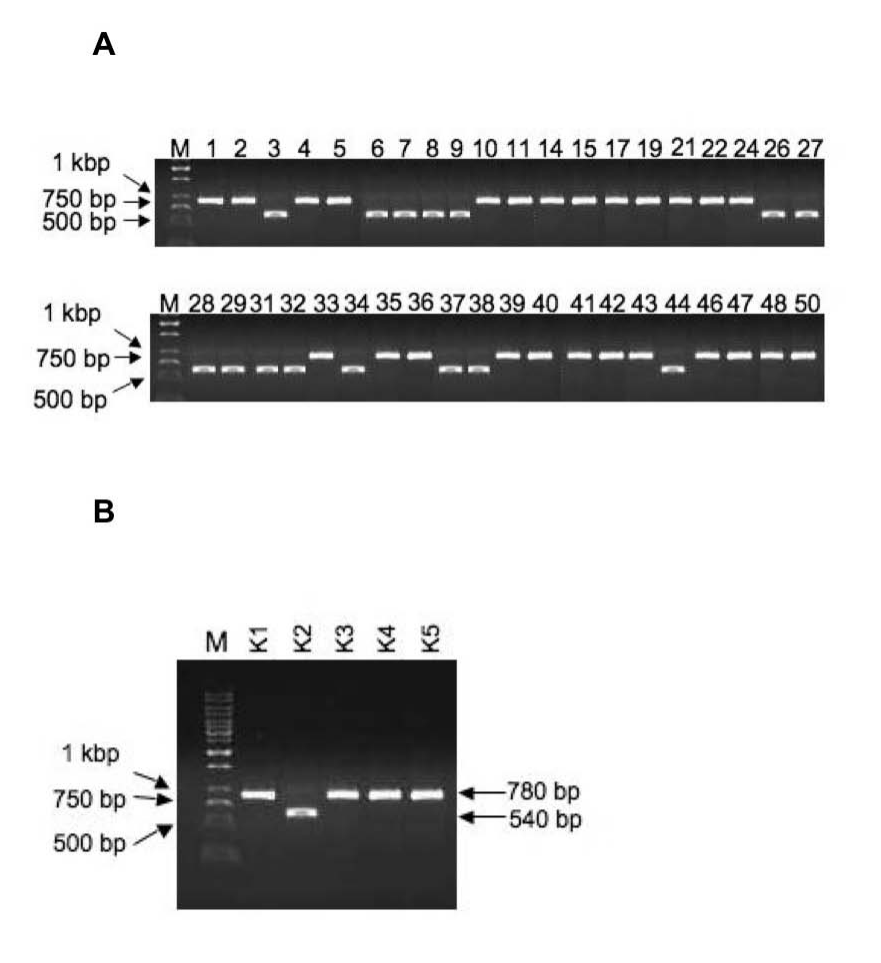
**DNA fingerprint analysis of specific scFv genes of positive clones**. scFv inserts were amplified from individual colonies of all positive clones. Amplification was done with Ex-Taq DNA polymearase enzyme using pCANTAB5-R1 and pCANTAB5-S6 primers. The amplified products were then analyzed on agarose gels. **A**. scFv encoding inserts of DNA of 40 diversified positive clones **B**. Only five restriction patterns of scFv encoding inserts of DNA were identified against five different clones (scFv K1 - K5). Molecular weights of DNA ladder markers (kbp) were indicated by M on the left.

To examine the integrity of the library after the final rounds of modified subtractive panning, 50 individual colonies in *E. coli *TG1 were randomly isolated and the presence of the scFv DNA insert in these clones was confirmed by using polymerase chain reaction (PCR) amplification; these amplified products were analyzed on 4% agarose gels (Figure 4A). Among these 50 colonies, 40 were positive. Only five restriction patterns were identified by the agarose gel electrophoresis; these patterns were confirmed by sequence data analysis. Among these five restriction patterns, four patterns contained inserts of the expected size (780 bp) fragments and the other pattern contained a low molecular weight DNA (540 bp) fragment (Figure 4B).

### Sequence analysis of scFv clones

**Figure 5:**
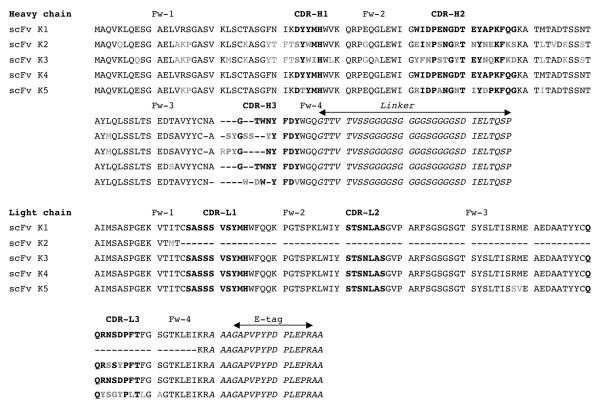
**Alignment of amino acid sequences of scFv K (1-5) antibody fragments**. Amino acid sequences were deduced from the nucleotide sequences. Positions of the respective complementarity determining regions for the variable domains of heavy chain (CDR H1-3) and light chain (CDR L1-3) were indicated by bold symbols. Framework (Fw) and CDRs as defined by Kabat et al. [44] were indicated. (Fw 1 - 4) were framework parts of antibody domains. To compare with scFv K1, amino acid differences in the other four clones were shown by gray symbols.

At least five unique genes (K1 - K5) were identified by nucleotide sequencing after the fifth round of modified subtractive panning. Table 2 showed the percentages of positive clones based on identical DNA sequences. Among all positive clones, K1 (19/40, 48%) and K2 (15/40, 38%) were identified as the highest populated group of clones after successful screening. Among the other positive clones, K3 was 10% (4/40) and K4 and K5 were each only 2%. Figure 5 showed amino acid sequences deduced from the obtained nucleotide data. All five sequences had different amino acid substitutions that were expected and mostly were in complementarity determining regions (CDR) [25]. The light chains of all clones were slightly different from each other, except scFv K2. For scFv K2, a light chain variable domain was partially synthesized together with an E-tag sequence. In the CDR area, the amino acid sequences shared relatively low sequence homology. Apparently, the degree of homology was observed higher in the variable region of the light chain (V_L_) domain compared with the variable region of the heavy chain (V_H_) domain. K1 and K4 differed by only one amino acid at position 93 in the V_H _domain.

### Production and characterization of nmAb-KT-specific scFv

All 40 individual positive clones after *E. coli *TG1 infection were reinfected into *E. coli *HB2151. In the nonsuppressor strain of *E. coli *HB2151, the amber stop codon between E-tag and g3 fusion protein was recognized as a stop codon, and a soluble scFv-E tag fusion protein was produced as a consequence. The expression of the scFv-E-tag fusion protein in the resultant *E. coli *HB2151 clones was induced by isopropyl β-D-thiogalactopyranoside (IPTG), and soluble scFv antibodies were secreted into the culture supernatants. After the final fifth round of modified subtractive panning, 80% clones (40/50) were positive with a strong binding affinity to nmAb-KT (Figure 3B). However, in regular panning after the fifth round of panning only 24% (12/50) were positive clones with low binding affinity (Figure 3A). The nmAb-KT-binding pattern of soluble scFv antibodies from these 40 positive clones of *E. coli *HB2151 was almost similar to that of the parent phage *E. coli *TG1 scFv antibodies, which was confirmed by using ELISA (Additional file 1, Figure S1). After screening, clones scFv K1 - K5 were identified, and soluble scFvs were purified from the periplasmic extract of *E. coli *HB2151 on an anti-E-tag affinity column.

### SDS-PAGE and Western blotting to check scFv expression

**Figure 6:**
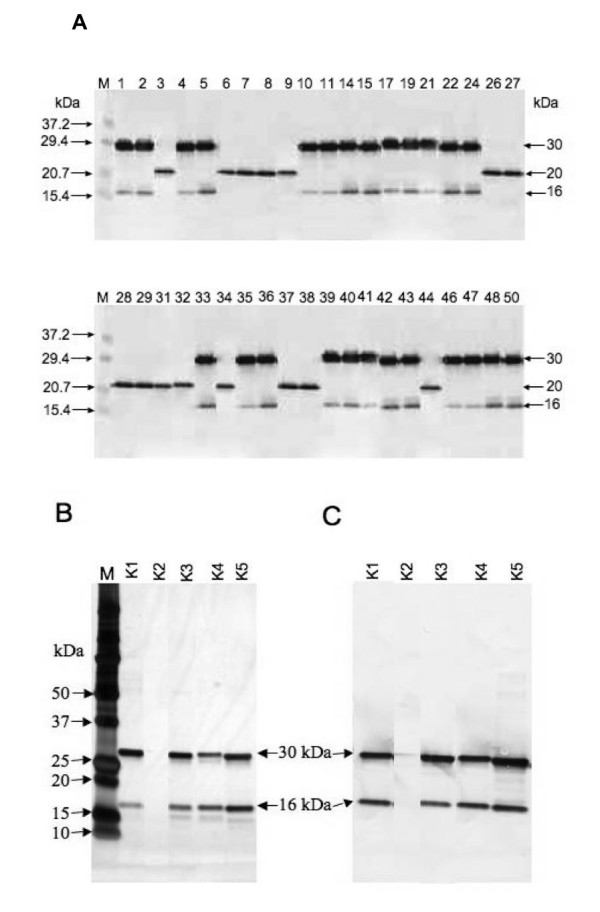
**SDS-PAGE and Western blot analysis of scFv expression**. **A**. Supernatant containing the soluble scFv antibodies for 40 diversified positive clones underwent SDS-PAGE and then Western blotting. **B**. Molecular weights of purified soluble scFv antibodies (K1 - K5) were detected by SDS-PAGE and were stained with silver stain. **C**. Western blotting of duplicate SDS-polyacrylamide gels was detected by probing with HRP-conjugated anti E-tag antibody. Molecular weights of marker proteins (kDa) were indicated by M on the left.

The culture supernatant without anti-E-tag column purification, an approximately 30 kDa (expected molecular weight (MW)) and two types of small (MW 20 kDa and 16 kDa) scFv-E tag fusion protein were expressed from the reinfected *E. coli *HB2151 (Figure 6A). There expressions indicated that all 40 positive clones originated from our original cDNA library constructed by pCANTAB 5 E vector using the E-tag sequence. Four clones of purified soluble scFv (K1, K3, K4 and K5) showed two strong protein bands (30 kDa and 16 kDa) by using sodium dodecyl sulphate-polyacrylamide gel electrophoresis (SDS-PAGE) and by Western blot analysis with HRP-conjugated anti-E-tag antibody (Figures 6B, C). Both proteins contained E-tag sequences, so Western blotting using HRP-conjugated anti-E-tag antibody detected bands. For the short sequenced scFv K2 clone, a 20 kDa E-tagged protein band showed before purification (Figure 6A), but after anti-E-tag column purification a protein band was not detected by using SDS-PAGE and Western blotting (Figures 6B, C).

### Functional characterization and antifungal activity of recombinant scFvs

**Figure 7:**
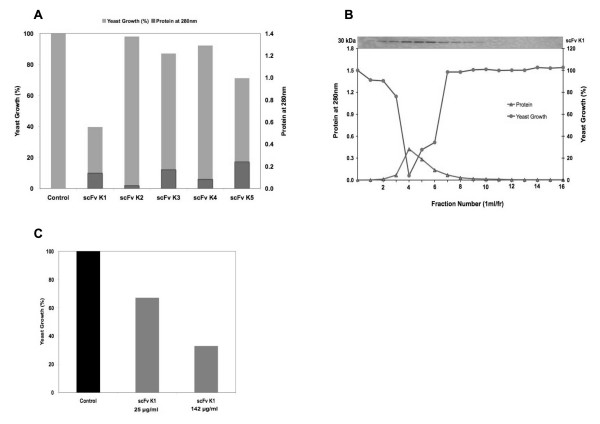
**Functional characterization of recombinant scFvs by measuring *in vitro *antifungal activity**. **A**. Biological activity of purified five clones scFv (K1 - K5). The effect of recombinant scFvs on the growth of *S. cerevisiae *A451 cells was measured by a cell suspension assay as described in Methods. Gray bars: Yeast Growth (%); black bars: Protein at 280 nm. **B**. Elution profile for scFv purification by using an anti-E-tag Sepharose column and the biological activity of the selected clone (scFv K1) against the growth of *S. cerevisiae *A451. Purified scFv K1 proteins per fraction (fr) were detected and visualized by SDS-PAGE and Western blotting with HRP-antiE-tag conjugate. A clear band of the expected size (30 kDa) for E-tag containing scFv was present in the purified elution fractions. **C**. Biological activity of scFv K1 against *C. albicans *ATCC 10231. For all cases fungal growth was measured at 600 nm. The results were means of duplicates.

*In vitro *antifungal activity of recombinant scFv antibodies was examined for non-pathogenic and pathogenic strains of fungal species *Saccharomyces cerevisiae *and *Candida albicans*, respectively. First, *in vitro *susceptibility of *S. cerevisiae *to scFv antibodies was examined by cell suspension assay as described in the Materials and Methods (Figure 7A). Among all scFv antibodies, scFv K1 was the most effective antifungal agent. Figure 7B showed the elution profile and biological activity of purified scFv K1 against the growth of *S. cerevisiae *A451. Next, to check the candidacidal activity of scFv K1 antibodies against *C. albicans *ATCC 10231, cell growth was also measured by cell suspension assay (Figure 7C). Table 3 showed the IC_50 _values of scFv K1 against *S. cerevisiae *A451 and *C. albicans *ATCC 10231 cells growth *in vitro*. Extensive studies by using our assay system showed that 2.20 × 10^-6 ^M and 3.83 × 10^-6 ^M were sufficient to produce 50% inhibition of growth of *S. cerevisiae *A451 and *C. albicans *ATCC 10231, respectively.

**Table 3:** IC_50 _values for the recombinant scFv K1 anti-idiotypic antibody against the cell growth of *Saccharomyces *and *Candida *species*

	IC_50 _(× 10^-6 ^M) for:	
	
Antibody	*C. albicans *ATCC 10231	*S. cerevisiae *A451
scFv K1	3.83	2.20

*IC_50 _values for recombinant scFv K1 against cell growth of *Candida *and *Saccharomyces *species were measured by using a cell suspension assay as described in the Methods.

### Kinetic parameters of selected clone scFv K1

The binding specificities and kinetic parameters of purified scFv K1, HM-1 and HYI killer toxin (HYI) were determined by using surface plasmon resonance (SPR) analysis. Biacore sensograms showed the degree of interaction between immobilized nmAb-KT and scFv K1, HM-1 or HYI (figure not shown). Kinetic data from SPR analysis showed that scFv K1 had a strong binding affinity for nmAb-KT, like HM-1, but HYI had no binding affinity for nmAb-KT. HM-1 analyte was used for the positive control and HYI analyte was used for the negative control experiment [26]. Table 4 showed the calculated values of *k*_on_, *k*_off_, *K*_a _and *K*_d_. The *K*_d _values of scFv K1 and HM-1 were 4.62 × 10^-8 ^M and 6.74 × 10^-9 ^M, respectively. Therefore, we can conclude that scFv K1 had strong reactivity with nmAb-KT, like HM-1, but not with HYI.

**Table 4:** Kinetic parameters for binding of scFv K1, HM-1 and HYI to nmAb-KT measured by SPR analysis

Ligand	Analytes	*k*_on _(× 10^4 ^M^-1 ^s^-1^)	*k*_off _(× 10^-4 ^s^-1^)	*K*_a _(× 10^7 ^M^-1^)	*K*_d _(× 10^-8 ^M)
nmAb-KT	scFv K1	1.02	4.71	2.17	4.62
nmAb-KT	HM-1	4.92	3.31	14.80	0.674
nmAb-KT	HYI	ND	ND	ND	ND

Ligand nmAb-KT was immobilized on the surface of a CM5 sensor chip, and scFv K1, HM-1 peptide or HYI peptide, were then injected over this immobilized surface in the presence of HBS-EP buffer. The affinity constant (*K*_a_) and dissociation constant (*K*_d_) were obtained from the ratio of the association (*k*_on_) and dissociation (*k*_off_) rate constants derived from global fits of response against time data to a 1:1 Langmuir binding model. *K*_a _= *k*_on_/*k*_off_; *K*_d _= *k*_off_/*k*_on_; ND, signals not detected. HM-1 analyte was used for the positive control and HYI analyte was used for the negative control experiment [26].

### Competitive binding of scFv K1 antibody with HM-1 to nmAb-KT

**Figure 8:**
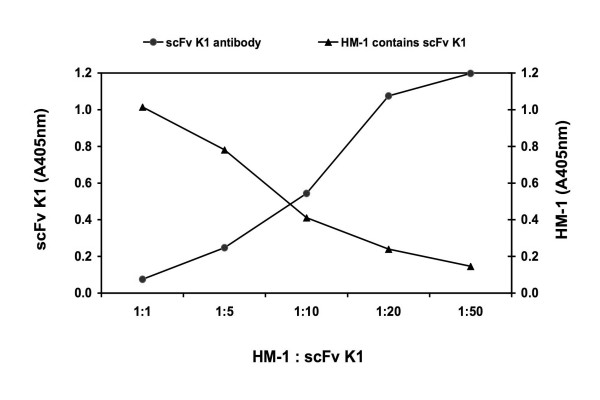
**Competitive binding analysis of the ability of HM-1 to inhibit the binding of scFv K1 antibody to nmAb-KT**. ELISA plates coated with nmAb-KT was incubated with increasing concentrations of the selected scFv K1 antibody as described in Methods. The results were means of duplicates.

To examine whether scFv K1 antibody compete with HM-1 to bind with nmAb-KT, a competitive binding ELISA was performed (Figure 8). When the scFv K1 antibody concentration was increased, the binding of scFv K1 antibody to nmAb-KT was also increased, but the HM-1 binding to nmAb-KT was simultaneously decreased. These results suggest that scFv K1 antibody clone was homologous in competition with HM-1 for binding to nmAb-KT.

## Discussion

Anti-idiotypic antibodies representing the internal image of some antigenic determinants have been proposed as surrogate vaccines [3,27], and antibodies conjugated with toxins have been proposed for immunotherapy of cancer [28]. In some cases, amino acid sequence homology exists between the protein antigen and the anti-idiotypic antibody variable region [29]. Antigenic mimicry is usually more functional than biochemical, and the functional mimicry of ligands of biological receptors by anti-idiotypic antibodies has been extensively studied [26,30]. To select a useful and specific scFv antibody for a target antigen, a high quality primary phage library is needed [31]. Importantly, the library must have a large functional size to match diverse antibody sequences to facilitate the selection of a variety of high affinity scFvs, and these scFv genes must be well expressed to allow the panning process. Previously, we established a large mouse scFv phage display library (2.8 × 10^10 ^CFU/ml), and we attempted to isolate antigen-specific scFvs by using nmAb-KT as a model antigen [32]. In this study, after proper selection of the library, phage-displaying anti-idiotypic scFv antibodies represented the internal image of the original antigen HM-1 and thus might be effective as a vaccine, avoiding immunization with a pathogen.

For traditional panning procedures, elution conditions are not same to recover a wide variety of specific phages from the immobilized antigens. Sometimes bound phages are not easily dissociated from the immobilized antigen because of tight antigen-antibody interaction and attachment with the solid support of the tubes or plates. Several methods have been reported to improve these problems to recover phage-displayed scFvs with the highest affinity, and some of these methods are applicable to the selection of anti-idiotypic antibodies [13,18,33-35]. Several elution methods that include the common acidic or basic conditions have been used to increase the stringency of selection [11,18]. More acidic or basic conditions sometimes may disrupt the phage particles, and hence scFvs may lose or change its functional characteristics. So, we were proceeding an alternative way that will be more susceptible to get a high affinity binders to the specific antigen. Therefore, during elution of the bound phages in modified subtractive panning, we selected neutral phosphate-buffered saline (PBS) solution (pH 7.0) supplemented with HM-1 that shared structural or functional similarities with the active site of phage-displayed scFv antibodies. As a result, elution with HM-1 facilitates and favors the panning process through competition of binding to immobilized nmAb-KT (Figure 1). This competition of binding accelerated the quick dissociation of scFv-containing phages from the immobilized antigen nmAb-KT with increased binding strength. The binding strength and specificity were analyzed by using ELISA with scFv antibodies against nmAb-KT. Figures 3A and 3B compared the bindings between antigen nmAb-KT and scFv antibodies obtained after using regular and modified subtractive pannings. The ELISA signals with soluble scFv antibodies from modified subtractive panning were much stronger than those from regular panning, indicating that the binding strength improved for elution with HM-1 in the selection procedure.

For further confirmation of the binding specificity of phage-displayed scFvs to antigen nmAb-KT after each round of panning, the number of recaptured phages was quantified. Figure 2 clearly demonstrated that after each round of panning the recaptured phages for modified subtractive panning were markedly higher than those for regular panning. Although the regular panning experiments were successful, they failed to enrich the phage clones sufficiently may be because of less dissociation of tightly bound scFv-containing phages from the solid support of the tubes or plates during selection.

To ascertain the scFv repertoire and the quality of the immunized antibody library, DNA segments encoding the scFv genes from 40 randomly selected positive clones (80%; 40/50) were identified after the fifth round of modified subtractive panning (Figure 3B). However, after the fifth round of regular phage display panning only 24% clones (12/50) were positive but with low binding affinity (Figure 3A). The 40 positive clones that had higher binding affinity were amplified by using the polymerase chain reaction (PCR), and the amplified products were analyzed on agarose gels and their DNA fingerprint patterns were compared (Figure 4A). The patterns of these clones were found to vary, indicating an excellent diversity in the immunized mouse scFv library. Nucleotide sequencing showed that at least five unique scFv genes were identified by using modified subtractive panning, i.e., clones scFv K1- scFv K5 were analyzed to deduce the amino acid sequences (Figure 5). DNA sequencing also confirmed that the highest population of the positive clones (48%, 19/40) was unique (scFv K1) with a higher binding strength to the antigen nmAb-KT. Also, the second highest populated group scFv K2 (38%, 15/40) had short DNA sequences in the V_L _domain compared with the other groups. DNA fingerprints of K1 and K3 - 5 were apparently identical (Figure 4B), although their amino acid sequences differed. Therefore, we produced at least five different anti-idiotypic scFv antibody clones of HM-1.

The scFvs, carrying the E-tag sequence and fused to the C-terminus, were purified by using an anti-E-tag affinity column and were analyzed on SDS-PAGE followed by Western blotting performed against HRP/anti-E tag conjugate. Silver-stained gel analysis of all purified scFvs showed a sharp, unique band (except scFv K2) of the expected MW of 30 kDa together with an unexpected band of lower MW (16 kDa); these bands were detected by Western blotting with the HRP/anti-E-tag conjugate (Figures 6B, C). The low MW 16 kDa bands had the E-tag sequence detected by Western blotting, indicating that they might be proteolytic cleavage derivatives of the original 30 kDa scFvs. Interestingly, scFv K2 expressed a 20 kDa smaller protein that also contained an E-tag sequence detected by Western blotting before column purification of the sample (Figure 6A). This 20 kDa scFv K2 had a partial V_L _region of the antibody, i.e., a residue of 16 amino acids instead of 99 amino acids that was confirmed from the DNA sequence data (Figure 5). This 20 kDa scFv K2 represented prematurely terminated translation products because of mutation. Purification of scFv K2 by using an anti-E-tag column was not possible to confirm by SDS-PAGE and Western blotting (Figures 6B, C). Therefore, the E-tag containing short scFv K2 (20 kDa) passed through the purification column without binding to the resin of the column that might be because of conformational changes due to partial lack of a light chain (V_L_) domain and might be because of cross-linking with other parts of the scFv antibody and therefore not having the open arm for binding with the antiE-tag sequence of the purification column.

scFv K1 that had the highest population (48%) of positive clones showed strong cytocidal activity against *S. cerevisiae *and *Candida *species compared with other scFvs (Figures 7A, B &7C). So, for further experiments we selected scFv K1 to characterize the functional activity and specificity. In this study, the antifungal activity was evaluated by cell suspension assay as described in the Materials and Methods. The scFv K1 antibody showed strong antifungal activity against *S. cerevisiae *and *C. albicans in vitro *with IC_50 _values 2.20 × 10^-6 ^M for *S. cerevisiae *A451 and 3.83 × 10^-6 ^M for *C. albicans *ATCC 10231. The anti-idiotypic scFv K1 had high affinities as judged by SPR. BIAcore analysis indicated *K*_d _= 4.62 × 10^-8 ^M for scFv K1, which was comparable to the HM-1 killer toxin (*K*_d _= 6.74 × 10^-9 ^M). Therefore, we concluded that nmAb-KT showed strong reactivity toward scFv K1, like HM-1, but not toward HYI. Furthermore, the binding of scFv K1 antibody to nmAb-KT was competitive with that of HM-1 (Figure 8), i.e., equimolar concentration of HM-1 inhibited the binding of scFv K1 antibody to the immobilized nmAb-KT antigen. Based on several factors, such as expression level, ELISA using nmAb-KT, BIAcore (*K*_d _= 4.62 × 10^-8 ^M) and fungal killing activity, scFv K1 was considered the most desirable scFv. So, scFv K1 is a candidate for future studies directed at killing of pathogenic fungal species using an scFv-drug conjugate or by development into a whole IgG format for complement-mediated and antibody-dependent cytotoxic pathways.

## Conclusions

In conclusion, we successfully obtained recombinant scFv antibodies with high specificity against nmAb-KT directly from the phage-displayed scFv library of a hyper-immunized mouse. Filamentous phage-displaying scFvs were selected against nmAb-KT and were enriched through five rounds of modified subtractive panning. The scFv antibodies generated after modified subtractive panning appear to have superior binding properties than for regular panning. We showed a simple modification of the elution condition in the phage-display panning protocol, and so make a large difference in determining success. The deduced amino acid sequences of CDRs might also help us to create other scFv fragments with even higher affinity and specificity for nmAb-KT and fungal cells by further mutagenesis study.

## Methods

### Bacterial and fungal strains

*E. coli *TG1 amber suppressor strain was used as the bacterial host to prepare phagemids and as the host for bacteriophage M13KO7. *E. coli *HB2151 non-suppressor strain was used to express soluble scFvs. *S. cerevisiae *A451 and *C. albicans *ATCC 10231 were a kind gift from Nippon Roche Research Center.

### Antigens, antibodies and conjugates

HM-1 was extracted from the broth culture of yeast *Williopsis saturnus *var. *mrakii *IFO 0895 and was purified as described previously [36,37]. HYI was prepared in our laboratory as previously described [38]. The purified nmAb-KT was prepared at the Technology Incubation & Transfer Ltd. (Saitama, Japan). M13KO7 helper phage, HRP-anti-E tag conjugate and an anti-E-tag Sepharose column were obtained from GE Healthcare UK Ltd., UK.

### Phage-displayed scFv library construction and phage rescue

HM-1-like anti-idiotypic scFv antibodies used in this study were produced according to the procedure of the Recombinant Phage Antibody System (GE Healthcare UK Ltd., UK.). Previously, a large mouse scFv phage display library 2.8 × 10^10 ^(CFU/ml) was constructed in our laboratory according to the manufacturer's protocol using a mouse scFv module and an expression module. Briefly, DNA fragments encoding V_H _and V_L _were amplified from reverse-transcribed mRNA by using PCR and were fused by a DNA fragment encoding a linker peptide. The assembled scFv fragments were ligated into the phagemid vector pCANTAB 5 E and then were transformed into competent *E. coli *TG1 cells so that the phage-displayed antibodies can be panned on immobilized antigen. Recombinant phages produced in *E. coli *TG1 were repeatedly panned against nmAb-KT and were screened by using conventional ELISA with nmAb-KT. The library was rescued by infection with helper phage M13KO7 for the next round of panning. The library size was calculated by counting the number of ampicillin-resistant colonies. Library quality was verified by determining the percentage of clones with inserts of appropriate size for a scFv gene. The diversity of the library was confirmed by sequencing randomly selected clones.

### Library selection on immunotubes against antigen nmAb-KT

#### A. Regular panning

The phage repertoire was panned by affinity selection using immunotubes (Nunc; Maxisorp) coated with antigen nmAb-KT [7,11]. The tubes were coated with nmAb-KT by incubating them overnight at concentration 10 μg/ml in PBS (pH 7.0) at 4°C. A large mouse scFv phage-display library containing 2.8 × 10^10 ^clones (CFU/ml) was used for the selection. The library stock was grown in log phase, was rescued with M13KO7 helper phage and was amplified overnight in 2× YT-AK (10 g bacto-yeast extract, 17 g bacto-tryptone, 5 g NaCl per litre of water (2× YT) containing 100 μg/ml ampicillin and 50 μg/ml kanamycin) at 37°C on a shaker. The phage was precipitated with 4% polyethylene glycol/0.5 M NaCl, was resuspended in 2× YT medium and was incubated with blocking buffer of PBS containing 4% skimmed milk at room temperature for 15 min to block nonspecific binding. After three times rinsing with PBS, phage particles were added to the immunotubes and were incubated on a shaker at 37°C for 2 h. After this incubation, unbound and nonspecifically bound scFv-phages of the tubes were washed away by rinsing the immunotubes 20 times with PBST (PBS containing 0.05% Tween 20) and then another 20 times with PBS. The bound phage particles were then immediately used to infect exponentially growing *E. coli *TG1 cells (OD at 600 nm = 0.5) for 1 h at 37°C. After each round of panning the infected cells were mixed with 10% glycerol and were then stored at -80°C. For the next round of panning, 3 ml of infected *E. coli *TG1 cell stock was added to 50 ml of 2× YT-AG (2× YT containing 100 μg/ml ampicillin and 2% glucose) medium and was grown to log phase. The culture was rescued with M13KO7 helper phage, was amplified, was precipitated, and was used for the next round of selection. Regular phage display panning was repeated five times as described above. The phage output from the final fifth round selection was propagated and 50 randomly selected *E. coli *TG1 colonies underwent ELISA.

#### B. Modified subtractive panning

To produce more effective antigen-specific recombinant anti-idiotypic scFv antibodies of HM-1 with superior binding strength to antigen nmAb-KT, a modified subtractive panning strategy was used for all five rounds of panning in the subsequent phage display experiment. The panning procedure was the same as the regular panning, except that an additional incubation step with HM-1 was added in each round of selection. To increase the elution stringency of the bound scFv-phages from the immobilized surface (nmAb-KT) of the tube, 10 μg/ml of HM-1 in PBS (pH 7.0) solution was added and was incubated at 37°C with a rotator shaker for 1 h. Specifically bound scFv-phages were eluted from the immobilized nmAb-KT and after the final fifth round of panning, the phages output were propagated, and 50 randomly selected *E. coli *TG1 colonies were selected for screening.

### Expression and purification of soluble scFvs

Soluble scFv fragments specific for the nmAb-KT antigen were produced by transfecting phages from positive clones identified by competitive ELISA into *E. coli *HB2151. This strain of *E. coli *recognizes the amber stop codon between the scFv and the gene III fragment. The procedures for infecting *E. coli *HB2151 cells and production of soluble antibodies were described in detail in the expression module of the recombinant phage antibody system (GE Healthcare UK Ltd., U.K.). Expression and periplasmic extraction of scFv were done as described by Yau et al. [12]. IPTG (1 mM) was used to induce scFv in exponentially growing cells (OD at 600 nm = 0.5) for 12 - 16 h at 30°C. The expressed products from whole cell extracts, supernatants, and periplasmic extracts of the specific colonies were analyzed to determine the location of accumulation of soluble antibodies. Recombinant soluble scFv antibodies were produced from the periplasmic extract of *E. coli *HB2151 cells, which were purified by using affinity chromatography with an anti-E-tag Sepharose column (Hi-Trap anti-E-tag column) according to the manufacturer's protocol. The concentration was determined spectrophotometrically at A_280 _nm 1.0 corresponding to 0.5 mg/ml scFv.

### ELISA screening of clones

Soluble scFv-based ELISA was done for screening. For both types of panning after the fifth round of selection, phages were rescued from single ampicillin-resistant colonies of infected *E. coli *TG1 cells [11,39]. The relative affinity and specificity of soluble scFvs were assessed against the antigen nmAb-KT as described by Clackson et al. [5]. Briefly, 96-well plates were coated with 10 μg/ml nmAb-KT in PBS overnight at 4°C, and were blocked with PBS containing 4% skimmed milk powder. Approximately 50 μl per well of soluble scFv supernatant from the overnight cell culture was added, was incubated for 2 h at room temperature and then was washed three times with PBST. The bound scFvs were detected by incubation with 8000-fold diluted of HRP-conjugated anti-E-tag antibody (GE Healthcare UK Ltd., U.K.) for 1 h at room temperature. The wells were washed again three times with PBST and 50 μl of 0.022% 2',2'-azino-bis(3-ethylbenzthiazoline-6-sulfonic acid [ABTS]/H_2_O_2 _substrate solution was added to each well by using citric acid. The absorption was measured at 405 nm.

### Fingerprint analysis of scFv genes and DNA sequencing

After the fifth round of modified subtractive panning, the diversity of selected scFv clones was analyzed by fingerprint analysis of the scFv genes of the positive clones. Plasmid DNAs from anti-idiotypic antibody producing clones were isolated from *E. coli *HB2151 by alkaline lysis and the scFv gene insert of individual clones was amplified by using PCR with pCANTAB5-R1 primer (5'-CCATGATTACGCCAAGCTTTGGAGCC-3') and pCANTAB5-S6 primer (5'-GTAAATGAATTTTCTGTATGAGG-3') and with Ex-Taq DNA polymerase enzyme (Takara). The amplified products were analyzed on agarose gels. The amplified V_H _and V_L _DNA portions in the plasmids were sequenced by using a CEQ 2000XL-DNA Analysis System with Dye Terminator Cycle Sequencing Quick Start Kit (BECKMAN COULTER, Inc.). The sequence alignment and manipulation were performed by using DNASIS program (Hitachi Corp.), and also the sequences were compared by using The Kabat Database system [40].

### SDS-PAGE and Western blotting

SDS-PAGE analysis was done as described by Laemmli U.K. [41]. Proteins were separated on 15% acrylamide gels, followed by staining of silver nitrate or Western blotting. For Western blotting, the purified periplasmic fraction of *E. coli *HB2151 culture underwent SDS-PAGE and was blotted on to a polyvinylidene fluoride membrane by using a semi-dry electroblotter. After blocking with 4% skimmed milk, the separated proteins were detected by HRP-conjugated anti-E-tag monoclonal antibody using Ez West Blue dye for HRP (Atto Corporation, Tokyo, Japan). A broad range pre-stained protein marker was used as a molecular weight marker.

### *In vitro *antifungal activity of anti-idiotypic scFv antibodies

A qualitative antifungal assay of purified scFv antibodies was done by using a cell suspension assay as described by Komiyama et al. [42]. Briefly, purified scFv antibodies were passed through an anti-E-tag Sepharose column at various concentrations in 80 μl of 20 mM glycine HCl, were neutralized with sodium phosphate buffer (pH 7.0), was mixed with 160 μl of YPD (1.0% bacto-yeast extract, 2.0% bacto-peptone and 2.0% glucose) medium containing 3 × 10^3 ^cells/ml of *S. cerevisiae *A451 and was incubated with gentle shaking at 30°C for 16 - 18 h. Glycine HCl neutralized with sodium phosphate buffer (pH 7.0) was used as a control. To measure the IC_50 _value, the concentration requiring 50% inhibition, different concentrations (10 - 80 μg/ml) of selected clone scFv K1 antibody was added to inhibit cell growth. The IC_50 _values were evaluated from semi-logarithmic graphs. To measure the biological activity against *C. albicans *ATCC 10231, the same procedure as described above was used, except the *Candida *concentration was 500 cells/ml.

### Affinity determination of selected scFv fragments by SPR analysis

The scFv K1 fragments, HM-1 (positive control) and HYI (negative control) were selected for determination of affinity to antigen nmAb-KT [26]. The binding kinetics of scFv K1 to immobilized antigen nmAb-KT were measured by using SPR analysis with a BIAcore × (BIAcore Uppasala, Sweden) according to the manufacturer's instructions. For the BIAcore experiments, nmAb-KT (30 μg/ml) was covalently immobilized on CM5 sensor chips using an amine coupling kit as described previously by Gruen et al. [43]. In this experiment, HBS-EP buffer (10 mM HEPES, 150 mM NaCl, 3 mM EDTA, 0.005% polysorbate 20 (pH 7.4)) was used, as the eluent buffer at a flow rate of 10 μl/min. The surface of the sensor chip was regenerated by pulse injection of 10 μl of 100 mM HCl (pH 1.2). The binding data were analyzed with the 1:1 Langmuir binding model in the BIAevaluation software (BIAcore).

### Competitive binding of scFv K1 antibody with HM-1 to nmAb-KT

ELISA plate was coated with 10 μg/ml of nmAb-KT and blocked with 4% skimmed milk powder in PBS solution for 1 h at 37°C. The scFv K1 antibody was applied in ratios of 1:1 to 1:50, followed by the addition of fifty microliters of HM-1 (50 ng/ml) as a competitor. Binding of scFv K1 was detected by HRP-conjugated anti-E tag antibody. Binding of HM-1 was also detected with anti-HM-1 rabbit serum followed by HRP-conjugated anti-rabbit IgG antibody (Promega, USA). Using ABTS-diammonium salt in citric acid, the absorption was measured at 405 nm.

## Abbreviations

ABTS: 2',2'-azino-bis(3-ethylbenzthiazoline-6-sulfonic acid; CDR: complementarity determining regions; ELISA: enzyme-linked immunosorbent assay; HM-1: HM-1 killer toxin; HRP: horseradish peroxidase; IPTG: isopropyl β-D-thiogalactopyranoside; nmAb-KT: killer toxin (HM-1) neutralizing monoclonal antibody; scFv: single chain fragment variable; SDS-PAGE: sodium dodecyl sulphate-polyacrylamide gel electrophoresis; V_H_: variable region of the heavy chain; V_L_: variable region of the light chain.

## Authors' contributions

MEK is the Ph.D. student at Department of Biochemistry, Niigata University of Pharmacy and Applied Life Sciences, under the supervision of TK. MEK performed all the practical experiment works and wrote the manuscript. SK is also another Ph.D. student of TK. SK, MM and YF helped to set up the BIAcore analysis, DNA sequencing and antifungal assay. TK supervised the whole works. All authors read and approved the final manuscript.

## Supplementary Material

Additional file 1 **Supplemental Figure Legend and Supplemental Figure S1**. Figure S1. Binding pattern of soluble scFv antibodies from *E. coli *TG1 and *E. coli *HB2151 to nmAb-KT. Click here for file
