# Supplementary material for: An improved phage-display panning method to produce an HM-1 killer toxin anti-idiotypic antibody
Source: BMC Biotechnol. 2009 Dec 14;9:99. doi: 10.1186/1472-6750-9-99 (PMC2801674; doi:10.1186/1472-6750-9-99)

Additional files

Additional file 1

SUPPLEMENTAL FIGURE LEGEND

Figure S1. Binding pattern of soluble scFv antibodies from *E. coli* TG1 and *E. coli* HB2151 to nmAb-KT. All 40 individual positive clones after *E. coli* TG1 infection were used to reinfect *E. coli* HB2151. The nmAb-KT binding pattern of soluble scFv antibodies from these 40 *E. coli* HB2151 infected clones was very near that of the parent phage (*E. coli* TG1) scFv antibodies.The binding pattern of these soluble scFv antibodies to nmAb-KT were measured by microtiter plate ELISA.

Supplemental Figure

Fig. S1

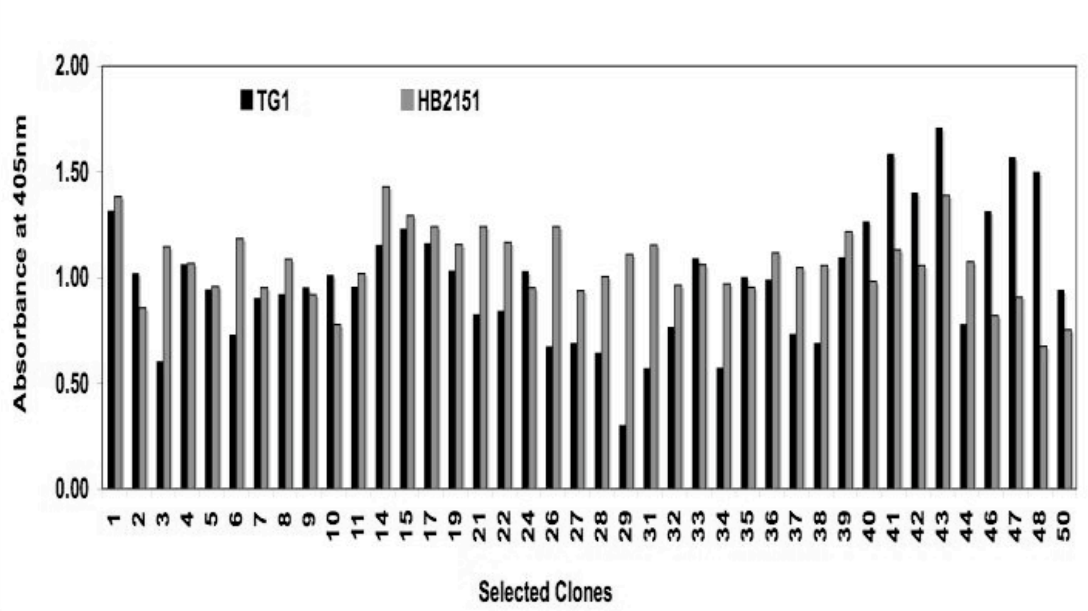

Supplement: Additional file 1 — Supplemental Figure Legend and Supplemental Figure S1. Figure S1. Binding pattern of soluble scFv antibodies from E. coli TG1 and E. coli HB2151 to nmAb-KT. [file 1472-6750-9-99-S1.PDF]
